# In Vitro Toxicity Studies of Bioactive Organosulfur Compounds from *Allium* spp. with Potential Application in the Agri-Food Industry: A Review

**DOI:** 10.3390/foods11172620

**Published:** 2022-08-29

**Authors:** Antonio Cascajosa-Lira, Pedro Andreo-Martínez, Ana Isabel Prieto, Alberto Baños, Enrique Guillamón, Angeles Jos, Ana M. Cameán

**Affiliations:** 1 Área de Toxicología, Facultad de Farmacia, Universidad de Sevilla, Profesor García González n 2, 41012 Seville, Spain; 2 Department of Agricultural Chemistry, Faculty of Chemistry, Campus of Espinardo, University of Murcia, 30100 Murcia, Spain; 3 DMC Research Center, Camino de Jayena, 82, 18620 Alhendín, Spain

**Keywords:** *Allium*, natural additive, bioactive organosulfur compounds, toxicity, in vitro

## Abstract

Organosulfur compounds (OSCs) are secondary metabolites produced by different *Allium* species which present important biological activities such as antimicrobial, antioxidant, anti-inflammatory antidiabetic, anticarcinogenic, antispasmodic, etc. In recent years, their use has been promoted in the agri-food industry as a substitute for synthetic preservatives, increasing potential accumulative exposure to consumers. Before their application in the food industry, it is necessary to pass a safety assessment as specified by the European Food Safety Authority (EFSA). This work reviews the scientific literature on OSCs regarding their in vitro toxicity evaluation following PRISMA guidelines for systematic reviews. Four electronic research databases were searched (Web of Science, Scopus, Science Database and PubMed) and a total of 43 works were selected according to predeterminate inclusion and exclusion criteria. Different data items and the risk of bias for each study were included. Currently, there are very few in vitro studies focused on investigating the potential toxicity of OSCs. Most research studies aimed to evaluate the cytotoxicity of OSCs to elucidate their antiproliferative effects focusing on their therapeutic aspects using cancer cell lines as the main experimental model. The results showed that diallyl disulfide (DADS) is the compound most studied, followed by diallyl trisulfide (DATS), diallyl sulfide (DAS), Allicin and Ajoene. Only 4 studies have been performed specifically to explore the safety of OSCs for agri-food applications, and genotoxicity studies are limited. More toxicity studies of OSCs are necessary to ensure consumers safety and should mainly be focused on the evaluation of genotoxicity and long-term toxicity effects.

## 1. Introduction

The name *Allium* is derived from the Greek word “aleo” defined as “to avoid” due to its strong odor [1]. The *Allium* genus includes about 600 to 700 species, with onion (*A. cepa*) and garlic (*A. sativum*) as the most well-known characteristic and edible species [2]. They were among the first domestic plants documented [3].

The main interest of these species lies in their organosulfur compounds (OSCs), which are secondary metabolites (e.g., ajoenes and thiosulphinates) with biological action and a distinctive smell of *Allium* species. When tissues are damaged, a cascade of enzymatic reactions by alliinase occurs, resulting in a degradation of initial compounds, and thus, sequentially, new highly reactive and biologically active compounds appear, giving rise to a great variety of byproducts [4]. These phytochemical compounds are biosynthesized for two main purposes: as a defense mechanism against biotic stresses, and as a mediator for pollination [1]. The *Allium* spp. are also well known for their multiple biological effects, such as antiviral, antimicrobial, antioxidant, antiprotozoal, antidiabetic, anticarcinogenic, antispasmodic, antimutagenic, anti-amnesic, antiasthmatic, anti-inflammatory, neuroprotective, hepatoprotective, hypotensive, immunomodulatory, hypoglycemic, as well as their prebiotic properties [2,4,5,6,7,8,9,10,11,12]. In relation to antibacterial activity, gram-positive bacteria seem to be more sensitive than gram-negative bacteria to these compounds. This could be due to lipopolysaccharides present in the outer membrane of Gram-negative bacteria. However, the mechanism of action is not yet sufficiently clarified, and it seems that it is associated with the inactivation of thiolic bacterial enzymes. Moreover, the mechanism of the anti-inflammatory activity of some OSCss can be associated with the inhibition of TNF-α-initiated secretion of pro-inflammatory cytokines from epithelial digestive cells. In addition, compounds such DADS, DATS and SAC inhibit the formation of inflammatory lipopolysaccharide by repressing NF-κB and MAPK signaling pathways [2].

Recently, Rochetti et al. [13] carried out an extensive phytochemical investigation of nine *Allium* species, highlighting a promising nutraceutical potential of these species. These properties give the *Allium* genus diverse uses in several areas of knowledge, and although traditionally this genus has already been used medicinally, in recent years its use has incremented in the agri-food industry [14]. Thus, *Allium* extracts and OSCss can be potentially used in food and feed sectors as a substitute for synthetic preservatives due to their significant antimicrobial properties [11,15]; nevertheless, their use in food is limited due to their organoleptic properties [1]. The use of OSCs and *Allium* extracts to control food spoilage have also been studied [16,17]. Several OSCs, such as propyl-propane-thiosulfinate (PTS) and propyl-propane thiosulfonate (PTSO) have a potential application in maize storage, mainly due to their antifungal and antimycotoxigenic activity. They could be used in concentrations in the order of parts per million to reduce up to 90% the concentrations of mycotoxins produced by *Fusarium* [18]. Moreover, due to their antioxidant and antibacterial activities, PTSO and PTS inserted in a synthetic matrix have been also proposed for usage in active food-packaging to better preserve salads [19,20]. As a consequence of their new applications, the potential accumulative exposure to consumers has increased, and a safe range of concentrations for their use in the industry should be established to reach the market and avoid risks for consumers (Table 1).

Furthermore, synthetic chemical additives, such as butylhydroxytoluene (BHT) and Butylhydroxyanisole (BHA), have shown problems regarding their safety, including carcinogenicity and sensitization in consumers [43]. For this reason, there is a call for replacement with natural and safe alternatives of natural origin, such as the bacteriocins [44] like Nisin (E234), a peptide produced from *Lactococcus lactis*, vegetable extracts such as citrus extracts rich in flavonoids [44] or essential oils (EO) with preservative properties, which have been reported to have antimicrobial and antioxidant properties [45]. In the case of garlic EOs, some authors demonstrated their preservation and antimicrobial capacities (including PTSO and PTS in its composition) [46,47]. However, important adverse effects produced by these compounds have also been described such as allergic reactions, gastrointestinal tract injury, weight loss, anemia, and toxicity to the liver, heart, and kidney [16]. Thus, garlic has been classified as a type I allergen.

Because of this all, to carry out all possible applications in the agri-food industry, it is necessary that OSCs pass a safety assessment as specified by the European Food Safety Authority (EFSA) [48,49,50,51]. This safety evaluation includes a wide array of tests both in vitro and in vivo. The “Guidance for submission for food additive evaluations” by EFSA [48] described the need of a toxicological evaluation in the following core areas: genotoxicity, toxicokinetics, toxicity comprising chronic, subchronic and carcinogenicity, developmental and reproductive toxicity. For the toxicological research, a tiered approach is followed, initially using fewer complex tests to obtain hazard data. These are then evaluated to determine if they are sufficient for risk assessment or, if not, for designing studies at higher tiers [48]. Therefore, in vitro studies are usually the initial step in the toxicological evaluation of any compound.

Thus, the objective of this work is to provide a systematic overview of the scientific literature of OSCs with antibacterial, antifungal, and other properties present in the *Allium* genus (Table 1) in regard to their in vitro toxicity evaluation. To achieve this purpose, the authors have followed the PRISMA 2020 guidelines for systematic reviews. This information could contribute to the safe use of these compounds in the agri-food sector. 

## 2. Materials and Methods

This research was performed according to the Preferred Reporting Items for a Systematic Review and Meta-analysis (PRISMA) statement [52]. The question to be answered was: Do *Allium* compounds have toxic activity in vitro?

### 2.1. Protocol and Registration

The protocol for the present systematic review was not registered in any Systematic Review and Meta-analysis database.

### 2.2. Eligibility and Exclusion Criteria

**Figure 1:**
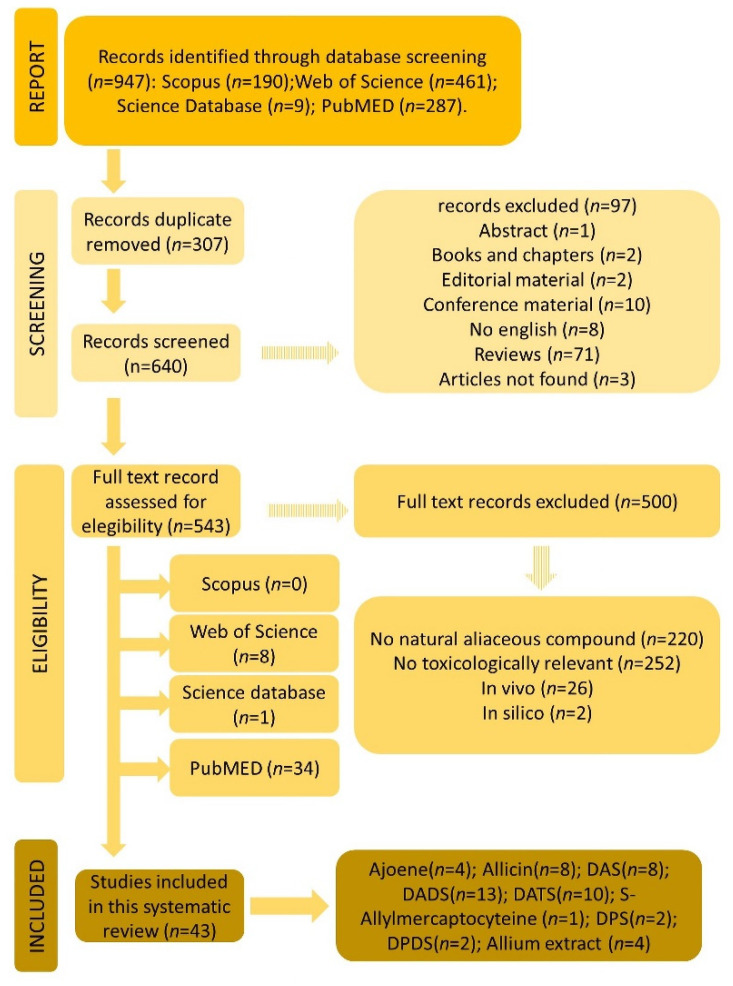
PRISMA flowchart of article selection.

International studies were considered. The eligibility criteria of the present systematic review were as follows: Inclusion criteria: (1) articles on *Allium* toxicity in vitro; (2) articles published prior to 21 September 2021; and (3) articles reporting comprehensive results and/or information on the field. Exclusion criteria: (1) unsystematic and narrative reviews; (2) articles published in a language other than English; (3) proceedings of conferences and dissertations; (4) books or book chapters; (5) editorial material; (6) articles dealing with *Allium* in vitro in which the test item is not a naturally occurring alliaceous compound (e.g., synthetically modified allicin) in the genus *Allium* or when the test has no toxicological relevance (e.g., protective effects of organosulfur compounds towards N-nitrosamine-induced DNA damage) as represented in the flowchart (Figure 1).

### 2.3. Information Sources and Search Strategy

The electronic research databases Web of Science, Scopus, Science Database and PubMed were searched on 21 September 2021. The search identified articles published from inception to 21 September 2021 inclusive. The Boolean strings chosen were: (“Propyl thiosulfinate oxide” OR “propyl-propane-thiosulfonate” OR “propyl propane thiosulfinate” OR “propyl-propane-thiosulfinate” OR “organosulfur compound*” OR diallyl* OR allicin OR alliin OR ajoene OR “dipropyl disulphide” OR “dipropyl sulphide” OR propiin) AND (toxicity OR cytotoxicity OR genotoxicity) AND (“in vitro” OR “cell line”). The searches included works published in all languages. The Web of Science database option search was “theme” in all databases. The Scopus database options search were: “title, abstract and keywords”. The Science Database option search was “all fields except full text (NOFT)” and the PubMed option search was “all fields”.

### 2.4. Study Selection

Once the selection criteria have been established, a three-step process was performed to review all records according to the eligibility criteria: first was reading the title, second, the abstract, and third, the entire text of the publication. The works obtained by the four databases were crossed with the EndNote X9 (Bld 12062) software to identify possible duplicates and to classify the works according to the exclusion and inclusion criteria. Two authors (PA-M and AC-L) formed the review team to implement measures to reduce random mistakes and bias at all review phases and independently examined titles, abstracts and full texts of the articles for possible addition. Conflicts on whether a given reference should be incorporated or not were determined through discussion. 

### 2.5. Data Extraction and Data Items

The data items included for data extraction were: Assays performed, experimental model, concentration ranges and time exposure, and main results. This data extraction form is presented in Table 2.

### 2.6. Risk of Bias in Individual Studies

Bias can be judged to be a systematic mistake that can lead to an underestimation or overestimation of the true result [53]. The risk of bias for each incorporated work was evaluated using The Methods Guide for Comparative Effectiveness Reviews [54]. The characteristics of bias considered are shown in Table 3. Each risk of bias question scored 2 (fully reported), 1 (partially reported) or 0 (unclear/not reported). Scores for each risk of bias question were added together to give a total score between 0 and 10. A score of 0–4 was considered a high risk of bias; 5–7 was considered a moderate risk of bias; 8–10 was considered a low risk of bias. The risk of bias assessment was performed by the reviewer team. Differences were resolved by discussion between the three reviewers (AC-L, AC and AIP).

### 2.7. Results Construction and Statistical Analysis

Compiled information and findings in the studies were examined independently by the reviewer team in order to find conflicts in the extracted data, should they be present.

## 3. Results

### 3.1. Search Results

The procedure of article selection is shown in the flow chart of Figure 1. The research database Web of Science returned 461 works; Scopus returned 190 works; Science Database returned 9 works and PubMed returned 287 works. The 947 works found by the four databases combined were crossed with the EndNote X9 (Bld 12062) software to identify possible duplicates. A total of 307 works were eliminated in this stage. After evaluating the abstract of each of the remaining articles, those that were associated with the subject of the study (43) were selected. According with the flow diagram (Figure 1), some articles were excluded since they had no toxicological importance, e.g., “Effect of allicin on promastigotes and intracellular amastigotes of *Leishmania donovani* and *L. infantum*”. It should be noted that some of the studies could be introduced in more than one exclusion group but the final criterion was agreed by discussion of the review team. Finally, a total of 43 works were found to be eligible for the present systematic review following the full-text eligibility assessment.

### 3.2. Study Characteristics and Results of Individual Studies

**Figure 2:**
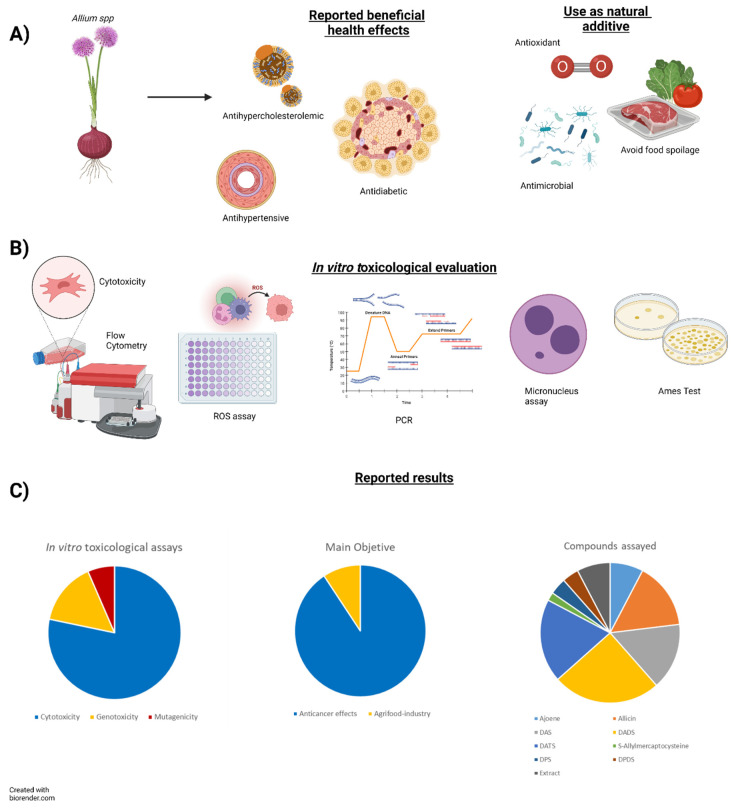
Graphical model: (**A**) reported beneficial health effects, (**B**) in vitro toxicological evaluation and (**C**) reported results.

The characteristics and main findings of the 43 selected articles in the present systematic review, such as Assays performed, experimental model, concentration ranges and time exposure and main results, are summarized in Table 2. Thus, the main tests carried out have focused on investigating cytotoxicity, cell apoptosis and ROS production against antioxidant assays. The MTT assay highlights how to determine the EC50 of these compounds in different cellular models. The flow cytometry is used for different determinations such as apoptosis, cell cycle, mitochondrial membrane potential and ROS. On the other hand, assays related to the mutagenicity and genotoxicity showed less interest. In this sense, Ames test, MN, comet assay and MLA have been performed. Regarding the experimental models, different cell lines have been used. Melanoma, lymphoma and gastrointestinal tract cells were the ones most often selected (Figure 2). The 43 selected articles were published between 1990 and 2021. According to the corresponding authors addresses, the articles were published in 19 different countries: 8 in China, 6 in Korea, 4 in USA, 3 in Spain, 3 in Poland, 2 in India, 2 in Taiwan, 2 in Germany, 2 in UK, 2 in Venezuela, 1 in Brazil, 1 in Israel, 1 in Egypt, 1 in Japan, 1 in Iran, 1 in France, 1 in Croatia, 1 in Turkey and 1 in Italy. The 43 included articles were published in 33 different journals, *Food* and *Chemical Toxicology* being the journals with most publications.

### 3.3. In Vitro Studies of OSCs from Allium spp. Focused on Safety Evaluation for Agri-Food Application

Of the 43 studies selected, only 4 have been performed specifically to explore the safety of OSCs from *Allium* spp. for agri-food applications [16,17,55,56] (see Table 2). Mellado-García et al. [17,18,19,20,21,22,23,24,25,26,27,28,29,30,31,32,33,34,35,36,37,38,39,40,41,42,43,44,45,46,47,48,49,50,51,52,53,54,55] studied the genotoxicity potential of PTSO and PTS using in vitro test battery including *Salmonella typhimurium* reverse mutation assay (Ames Test), The cytokinesis-block micronucleus cytome assay (MN) and single cell gel electrophoresis assay (comet assay). Both compounds reported negative results by Ames Test and genotoxic effects were described in similar concentration for PTSO and PTS by MN assay. However, MLA and comet assays showed contradictory results between these compounds. No cytotoxicity or mutagenicity of DPS, DPDS, and mixtures were reported by Llana-Ruiz-Cabello et al. [16]. In relation to polymer films of *Allium cepa* L., no induction of MN was observed, although the HTP films showed signs of mutagenicity by the Ames Test [56].

**Table 2:** Overview of the studies reporting the in vitro toxicological evaluation of OSCs from *Allium* spp.

*Allium* Products (Pure Compound or Extract)	Assays Performed	Experimental Model	Concentration Ranges and Time Exposure	Main Results	Reference
Ajoene Allicin	Survival by MTT assay Total cell protein	FS4 BHK21 BJA-B	5–20 µg/mL for 48 h.	EC_50_(ajoene): FS4 (36 µM) > BHK21 (30 µM) > BJA-B (12 µM). ED_50_ (ajoene): FS4 (450 fmol/cell) > BHK21 (190 fmol/cell) > BJA-B (85 fmol/cell). ED_50_(allicin): FS4 (720 fmol/cell) > BHK21 (430 fmol/cell) > BJA-B (185 fmol/cell).	[57]
Ajoene	Metabolic activity by MTT assay Cell death by trypan blue assay Levels of GSH, GSSG and acidic aminoacids Glu and Asp	BJA-B cells	173 and 82 nmol/mL for 0–6 h. 385 and 150 fmol/cell for 48 h.	In both experiments, ↓ cell viability in a dose and time dependent manner. In the first minutes after exposure, GSH decreased and GSSG increased. The further course strongly depended on the dose. The Glu pool showed an immediate increase and in a later stage decreased. The Asp pool showed the contrary pattern.	[58]
DDS DAS	Clonal survival Mass growth rate. Anchorage-Independent Growth.	Control and differentiated HT29 cells	1–3 µg/mL DDS 24 h 100–300 µg/mL DAS 24 h after which the cells were incubated in fresh medium for 10–12 days. Cells were exposed to 24-h intervals for seven days.	Dq (concentration at which no cell killing occurs) 0.10 ± 0.03 µg/mL for DAS and was not found for DDS. D37 (dose required to reduce survival from 100% to 37%) are 2.93 ± 0.14 µg/mL for DAS and 164 ± 12 µg/mL for DDS.	[59]
DAS, DADS	Clonal survival Induction of chromosome aberrations Induction of SCEs	CHO	100–600 µg/mL DAS 2–10 µg/mL DDS	Cytotoxicity DADS > DAS. Both compounds induced chromosome aberrations and SCEs (DADS > DAS) +S9: reduction of the induction of SCEs by both compounds, and enhanced the generation of aberrations by DADS.	[60]
DAS, DADS, DPS, DPDS	Ames test	Ames test: *Salmonella typhimurium* strains TA98 or TA100	Ames test was performed with S9 and microsomes from DAS-, DADS-, DPS-, DPDS-treated rats (1 mmol/Kg)	DAS, DPS, DPDS: increased activation of BaP, CP, N-PiP and PhIP, while DADS only increased mutagenicity of PhIP. In contrast, some OCS inhibited the mutagenicity of different mutagens, while other enhanced it.	[61]
DAS, DADS, garlic extract	Cytotoxicity by MTT assay Western blot analysis of Bcl-2, Bax and p53 expression Northern blot analysis Apoptosis by acridine orange staining	p-53-wild type H460 and p-53-null type H1299 non-small cell lung cancer cells (NSCLC)	DAS and DADS (0–25 µM) and garlic extract (0–200 µg/mL) for 1 h	The cell growth was significantly inhibited by DAS and DADS and slightly inhibited by garlic extract. The OSCs compounds and garlic extract have apoptotic potential on lung cancer cells, and the mechanism was regulated through p53-dependent or p-53 independent related Bax/Bcl-2 dual pathway.	[62]
DADS	Cell viability and apoptosis by flow cytometry Oxidative stress (carbonylated proteins, MDA) Western blot analysis	SH-SY5Y	50 µM for 12 and 24 h 50 µM up to 2 h	Blockage in G2/M phase DADS induced a ROS-mediated activation of JNK/c-Jun pathway in neuroblastoma cells, and this activation led to apoptosis.	[63]
DADS	Survival by MTT assay Apoptosis by flow cytometry Cell signals by western blot analysis of phosphorylated forms of GSK-3β and Akt, and p85a PI3K Free radical levels and membrane lipid peroxidation	N18D3	10–200 µM for 2 h. 10, 25 µM for 2 h pretreatment and treated with 100 µM H_2_O_2_ for 30 min. 25, 100 µM for 2 h treatment with and without post-treatment of 100 µM H_2_O_2_ for 30 min.	Cellular viability was not affected up to 25 µM DAS. ↑ apoptotic cells at 100 µM of treatment and at 25 µM of pretreatment versus H_2_O_2_ treatment in these cells. ↑ the expressions of p85a PI3K, phosphorylated Akt and phosphorylated GSK-3 in N18D3 cells pretreated with 25 µM (2 h) and subsequently exposed to 100 µM H_2_O_2_ (30 min). Treatment with 100 µM reduced these biomarkers in N18D3 cells. ↑ the levels of free radicals and membrane lipid peroxidation a concentration-dependent manner.	[64]
Allicin	Cell proliferation by MTT assay Morphological apoptosis DNA fragmentation assay Cell cycle by flow cytometry Caspase-3 determination Expression of Cyt *c*, Bax and AIF by Western blot analysis	AGS	5–100 µg/mL for 6, 12, 24 and 48 h.	Allicin caused inhibition of cellular growth in a concentration- and time-dependent manner. DNA fragmentation and morphological changes (degeneration of neuritis, shrinkage of cell bodies and condensation of nuclei) in cells exposed to 5–20 µg/mL for 24 h. 45.2% apoptotic cells. ↑ in the sub-G1 DNA content. ↓ in the proportion in cells of S phase after exposure to 20 µg/mL of allicin for 24 h. Allicin results in the release of Cyt c and in increase of mitochondrial Bax protein level. Caspase-3 activation and cleavage of PARP were not detected.	[65]
Ajoene	MTS/PMS chromogenic assay Cell adhesion assay	B16/BL6 HT-29 A549 MDA-MB-231 PANC-1 SKBR-3 NIH 3T3 3T3/HER2 Splenocytes B16/BL6-LEC1	10–100 µM for 24 h 1–100 µM for 24 h	IC_50_(B16/BL6) = 18 µM IC_50_(HT-29) = 19 µM IC_50_(A549) = 41 µM IC_50_(MDA-MB-231) = 7 µM IC_50_(PANC-1) = 38 µM IC_50_(SKBR-3) = 19 µM IC_50_(NIH 3T) = 17 µM IC_50_(3T3/HER2) = 9 µM IC_50_(Splenocytes) ≥100 µM B16/BL6 29% inhibition at 10 µM	[66]
DAS, DADS, DATS	Cell viability by trypan blue exclusion assay Wright staining and ApopTag assay for apoptosis ROS production Intracellular free [Ca^2+^] by Fura-2 assay mRNA expression of β-actin, bax, bcl-2, calpastatin phosphorylation of stress kinases analysis Western Blot analysis analysis Mitocondrial membrane potential Caspase 3 and 9 activity GST activity	T98G U87MG	100 µM DAS 100 µM DADS 25 µM DATS for 24 h	The three garlic compounds induced cytotoxic effects via ROS production, increase in endoplasmic reticulum (ER) stress, decrease mitochondrial membrane potential, activation of stress kinases and cysteine proteases.	[67]
Allicin	*In vitro* tubulin polymerization assay and image analysis	NIH-3T3	0.2–25 µM	Depolymerizing effect of allicin in a concentration-dependent manner until 25 µM. Disruptive effect of allicin increases with the duration of incubation.	[68]
Ajoene (≥98%)	Cell viability by MTS assay Apoptosis by flow cytometry Microscopic evaluation	B16F10	1, 5, 10 µmol/L for 24 h	5 and 10 µmol/L ↓ cell viability and this cytotoxic effect was not prevented by the addition of mevalonate or GGPP. Ajoene (5 µmol/L) in combination with atorvastatin (0.1 mmol/L) or pravastatin (0.1 mmol/L) ↓ cell viability in a synergistic way. Apoptosis observed by diminution in cell volume, condensation of cytoplasm.	[69]
DADS	Cell viability by MTT assay Apoptosis by fluorescence microscopy and flow cytometry p-ERK and ERK protein levels by Western blot analysis	MCF-7	50–400 μmol/L for 24 h. 200 μmol/L for 6, 12, 24 and 48 h.	↓ cellular viability and ↑ apoptosis in concentration-dependent manner. These effects were observed mainly from 200 μmol/L of treatment. Inhibition of ERK and activation of SAPK/JNK and p38.	[70]
DAS	Cell viability by trypan blue exclusion assay ALP and LDH assays ROS generation Apoptosis by flow cytometry analysis Cell cycle analysis DNA fragmentation analysis Immunoblot analysis of caspase-3, NF-κB, ERK-2	Colo 320 DM	5–75 µM for 0–24 h 50 µM for 6, 12 h 50 µM for 12 h 50 µM for 12 h	50 µM ↓ cell viabilityALP and LDH decreased with time. ROS increased.~55% apoptosis. Cell cycle arrest at G2/M Oligonucleosomal-laddering, characteristic of apoptosis.Higher protein expression of caspase-3 and NF-κB and lower expression of ERK-2.	[71]
DADS	MTT assayApoptosis by flow cytometry, DNA fragmentation assay and morphology analysisWestern blot analysis of the expression of phosphor-MAPKs (ERK, p38)	HL-60	1.25–20 mg/L for 96 h 5–20 mg/L for 24 h 10 mg/L for 24 h	10 mg/L ↓59.6% cell viability Apoptosis was evidenced in a concentration-dependent manner by different assays. Inhibition of ERK and activation of p38	[72]
DATS	MTT assay Apoptosis by flow cytometry Expression of Bcl-2, Bax, Bcl-xL/Bcl-xS, Cyt c, caspase 9 and poly(ADP-ribose) polymerase by western blotting. Activity of caspase 3	A375 M14	5–60 µM for 24, 48, 72 h Exposure to IC_50_ for 72 h: A375 11.7 µM and M14 14.1 µM IC_50_ for 24, 48, 72 h IC_50_ for 16, 24, 36, 48 h	IC_50_(A375) = 11.7 µM IC_50_(M14) = 14.1 µM ↑ percentage of apoptosis Reduced Bcl-2 and Bcl-xL expression Increase in caspase-3 activity with time	[73]
n-DADS s-DADS	MTT assay Cell cycle analysis Apoptosis by flow cytometry and by fluorescence microscopy after staining with Hoechst 33,258	MCF-7	0.01–2.00 mmol/L 48 for 72 h. 0.01, 0.05, 0.25 mmol/L for 48 h. 0.05 mmol/L for 48 h, microscopy	s- and n-DADS present concentration- and time-dependent inhibitory effects and similar cytotoxicity in MCF-7 cells. Apoptosis from 0.01 mmol/L for 48 h. The percentages of cells in G0/G1-, S- and G2/Mphase did not differ from each other.	[74]
DATS	Cell viability by trypan blue assay ROS by fluorescence microscopy and flow cytometry Mitochondrial ROS levels assay Immunoblot analysis	MDA-MB-231	10–100 μM for 16 h 10–80 μM for 1 h 10, 50 μM for 1 h 10–100 μM for 16 h	Apoptotic cell death in concentration- and time-dependent manner was observed with cell shrinkage and cytoplasmic membrane blebbing. ↑ ROS with activation of ASK1 and a downstream signal transduction JNK (C-Jun N-terminal kinase)-Bim pathway at 50–80 μM.	[75]
DAS	Cell viability, cell cycle and apoptosis by PI staining by flow cytometric assay DNA damage by Comet assay and DAPI staining Flow cytometric assay for the production of Ca^2+^ and the level of mitochondrial membrane potential Western blot of apoptotic associated proteins Microarray assay	HeLa	25–100 µM for 24 h 75 µM for 0–2 h 75 µM for 0–72 h 5 μM DAS for 24 h	DNA damage and fragmentation. Induced apoptosis and decreased the viability in concentration- and time-dependent manner. Induced cell cycle arrest increasing G0/G1 cell population and decreasing G2/M and S cell population. Decreased levels of mitochondrial membrane potential and promoted the levels of Ca^2+^ DAS promoted the levels of Fas, FasL and caspase-8, Bax, cytochrome c, Apaf-1, Bid, caspase-9 and -3. 28 genes were expressed at least by 2-fold compared with the untreated control cells.	[76]
DATS	Comet assay Apoptosis and ROS by flow cytometry Immunoblotting for phosphoolorect-ERK1/2 (P-ERK1/2	PC-3 cells transfected with the plasmid encoding p66ShcS36A or an empty pcDNA3.1 vector	After 24 of transfection, cells were treated with DATS (0–40 µM)	DATS increased p66Shc phosphorylation at serine 36, which was abolished by JNK inhibitor, and DATS-induced ROS formation was abolished in cells expressing p66ShcS36A variant. In cells expressing this variant, DATS-induced Akt dephosphorilation was reduced. The signaling pathway with P66Shc could be indispensable for DATS-induced prostate cancer cell death by modulating the Akt activity and ROS generation.	[77]
DATS	ROS by flow cytometryProtein levels by ImmunoblottingLabile iron poolDNA damage by comet assay and microscopy	PC-3	40 µM for 4 h 40 µM for 12 h	DATS-mediated increase in labile iron pool is regulated by JNK1 but not JNK2. Ferritin degradation in PC-3 cells treated with DATS is controlled by JNK1. DATS-induced increase in ROS formation is JNK1-dependent. Iron is not involved in DATS-induced cell death. DATS-induced DNA damage is not ameliorated by iron chelation.	[78]
DATS	Cell viability by MTT assay Apoptosis by DAPI staining ROS and Mitochondrial membrane potential by flow cytometry Caspase-9 and -3 activities Apoptosis associated proteins by Western blotting	Primary colorectal cancer cells	10–40 µM for 24 h 20 µM for 6,12 h 20 µM for 24 h	Viability inhibition in a concentration-dependent way. Apoptosis induction. Nuclear shrinkage/condensation and nuclear fragmentation. ROS production induction and decreased level of mitochondrial membrane potential. Activation of caspase 9 and 3. Increased protein levels of cytochrome c, caspase -9 and caspase-3.	[79]
DATS	Cell survival by sulforhodamine B assay ROS by flow cytometry Protein level by immunoblotting	PC-3 PNT1A	40 µM for 24 h	PNT1A cells are more resistant to cytotoxic effects than PC-3 cells. In these cells, reduction of induced p66Shc hosphorylation and ferritin degradation, reduction Akt inactivation, and ROS generation was nearly abolished in PNT1A cells.	[80]
*Allium sivasicum* aqueous extract	Cytotoxicity by Trypan blue exclusion assay and MTT assay Apoptosis by flow cytometry	MCF-7 MDA-MB-468 MDA-MD231	10–100 µg/mL, 48 h MCF-7 21 ± 1.4 µg/mL MDA-MB-468 22 ± 1.4 µg/mL MDA-MB-231 24 ± 1.3 µg/mL (24 h for all)	IC_50_(MCF-7) = 21 ± 1.4 µg/mL IC_50_(MDA-MB-468) = 22 ± 1.4 µg/mL IC_50_(MDA-MB-231) = 24 ± 1.3 µg/mL ↑ percentage of apoptosis	[81]
S-Allylmercaptocyteine	Cell proliferation by [^3^H] thymidine incorporation assay DNA fragmentation assay Free SH groups Cell cycle by flow cytometry Cytotoxicity by MTS assay	HEL OCIM-1	0.02, 0.05, 0.1, 0.25 mM 24 h in HEL cells 0.05 or 0.1 mM for 2 days 0.1, 0.25, 0.5, 1 mM for 1,2, 3 days 0.25 mM for 6 h and 0.1 mM for 72 h 0.002–2 mM	Significant reduction in [^3^H] thymidine incorporation Signs of DNA fragmentation Initial increase of free SH groups followed by progressive decrease with extended incubation Accumulation of cells in G2/M phase OCIM-1 more sensitive. LD50 (HEL) = 0.1 mM and LD50 (OCIM-1) = 0.046 mM	[82]
Allicin	Cell proliferation by MTT assay Apoptosis and cell cycle by flow cytometry	SGG-7901	Not revealed Apoptosis: 3 mg/l for 12, 24, 48 h Cell cycle: 3, 6, 12 mg/L for 24 and 48 h	Growth inhibition in a concentration-dependent manner Increased apoptosis Cell cycle arrest in G2/M	[83]
DADS	Cell viability by MTT assay Apoptosis by phase contrast microscopy and flow cytometry	ECA109 L02	10–60 µg/mL for 24 h 20–80 µg/mL for 24 h	Cell viability inhibition in a concentration-dependent manner in ECA109. Less toxic in L02 Membrane blebbing and formation of apoptotic bodies. Cellular shrinkage. Apoptosis induction in a concentration-dependent manner	[84]
DADS	Cell viability by MTT assay Cell cycle and apoptosis by flow cytometry PCR to investigate G2/M phase relative molecular pathway Protein expression by Western blot	ECA109 L02	10–60 µg/mL for 24–72 h 20–60 µg/mL for 24 h	Cell viability Inhibition in a concentration-dependent manner. Apoptosis induction in a concentration-dependent manner. G2/M phase arrest. Upregulated levels of p21 and p53 Protein levels of caspase-3 and cleaved caspase-3 upregulated in a concentration-dependent way. Induced apoptosis through upregulation of Bax mRNA, downregulation of Bcl-2 mRNA and a shift of Bax/Bcl-2 ratio. Expression levels of MEK1 and ERK1/2 did not change, but p-MEK1 and p- ERK1/2 decreased	[85]
PTSO	Ames test MN test MLA assay comet assays (with and without Endo III and FPG enzymes)	*Salmonella typhimurium* strains L5178Ytk+/- Caco-2	5–100 µM for the different assays, depending on the viability of the cells (Trypan blue exclusion test)	PTSO was not mutagenic in the Ames test, although it was weak mutagenic in the MLA assay after 24 of treatment (2.5–20.0 µM). The parent compound did not induce MN on mammalian cells, although in presence S9, induced positive results (20 µM). PTSO did not induce DNA breaks or oxidative damage in the comet assays.	[17]
DPS, DPDS, and mixtures	Cell viability by PC, NR, MTS ROS, GSH Morphology study Ames test	Caco-2 cells *S. typhimurium* strains	0–200 µM for 2, 4, 8 h	No cytotoxicity or mutagenicity and no significant adverse effects were reported. ROS scavenger activity was observed for both compounds.	[16]
Allicin	Cell viability by MTT assay Apoptosis by Hoechst staining and flow cytometry Expression levels of apoptosis-associated proteins by western blotting	MGC-803 BGC-823 SGC-7901	0.5–10 µg/mL for 48 h 1 µg/mL for 12, 24 and 48 h 0.01–10 µg/mL for 48 h	Cell viability is affected in a concentration and time-dependent manner. Apoptosis induction Enhanced expression levels of cleaved caspase 3	[86]
DAS, DADS, DATS	Cytotoxicity assay by cell counting kit-8Protein expression by western blottingCaspase-8 and 9 activityImmunofluorescence analysisLuciferase reporter assayRT-PCR	BC3BCBL1HBL6BC2Ramos DG75	1–50 µM for 24 h	DAS and DADS slightly decreased viability DAT: IC_50_(BC3) = 13.7 ± 0.8 IC_50_(BCBL1) = 15.5 ± 1.0 IC_50_(HBL6) = 17.7 ± 0.6 IC_50_(BC2) = 14.6 ± 0.4 IC_50_(Ramos) = 43.4 ± 1.4 IC_50_(DG75) = 48.0 ± 0.9 Apoptosis by activation of caspases Suppression of NF-κB signaling	[87]
PTS	Ames test MN assay MLA assay comet assays (with and without Endo III and FPG enzymes)	*S. typhimurium* strains for Ames test; L5178Ytk+/− cells for MN and MLA assays; and Caco 2 cells for comet tests	0–280 µM for the different assays, depending on the viability of the cells (total protein, NRU, MTS)	Not mutagenic neither in the Ames test nor in MLA. Genotoxic effects were reported in the MN test at the highest concentration assayed (17.25 µM) without S9, and also its metabolites (+S9, from 20 µM). ↑ breaks damage on CaCO_2_ cells at the highest concentration tested (280 µM) but it did not induce oxidative DNA damage.	[55]
DATS	Cell viability by MTT assay Cell cycle and apoptosis by flow cytometry Protein expression by western blot Nuclear morphological changes ROS and MMP	AGS Chang liver cells	0–50 µM for 0–24 h 50 µM, 0–24 h	Concentration- and time-dependent decrease of cell viability in AGS cells. No effect on Chang liver cells. In AGS cells DATS induced G2/M arrest and apoptosis by blocking cell cycle into G1 phase, mitotic arrest, caspase-dependent apoptosis, and ROS-dependent AMPK activation	[88]
DATS	Cell viability by trypan blue exclusion assay Clonogenic assay ROS Expression of DR4 and DR5 by flow cytometry Immunocytochemistry Apoptosis by flow cytometry Immunoblotting.	U87MG A172 U343 T98 G	25–50 µM for 30 min 25 µM 24 h 5–50 µM for 24 h and 25 µM for 0–24 h	Up-regulated DR5 receptor expression, and enhanced TRAIL-induced apoptosis through the downregulation of anti-apoptotic protein Mcl-1 and the upregulation of DR5 receptors through actions on the ROS-induced-p53	[89]
Allicin	Cytotoxicity by MTT assay Cell proliferation and colony formation assays Protein expression by western blot analysis Gene expression by RT-qPCR Caspase activity Morphology study Apoptosis by flow cytometry	U251	15–90 µg/mL for 24 h. 5–90 µg/mL for 24, 48, 72 h. 30, 60 µg/mL for 48 h 30, 60 µg/mL 30, 60 µg/mL, 24 h 30, 60 µg/mL, 48 h	Cytotoxic effect in a concentration-dependent manner and nuclear morphology changes in U251 cells. IC_50_ = 41.97 µg allicin/mL for 24 h. Increased apoptosis Morphological changes of apoptotic cells (condensation of chromatin, nuclear fragmentation) Proliferation inhibition ↑ caspase-3, -8 and -9 activities and Fas/FasL and Bax mRNA expression levels. ↓ Bcl-2 expression levels in a dose-dependent manner. ↑ the activation of both intrinsic and extrinsic apoptosis signaling pathways in U251 cells.	[90]
DAS	Cell viability by MTT assay The extend of lipid accumulation ROS by flow cytometry qRT-PCR of inflammatory genes	3T3L1 RAW 264.7	100 mM ethanol and treated with 50–500 µM DAS for 24 and 48 h.	↑ viability in ethanol-exposed 3T3L1 cells treated with 200–500 µM for 24 h and 50–500 µM for 48 h. ↓ ROS production, reduces expression of pro-inflammatory cytokines, and enhance anti-inflammatory cytokine production in ethanol-exposed 3T3L1 cells treated with 50–100 µM for 24 or 48 h. 100 µM for 24h ↑ expression of M2 phenotype- specific genes in ethanol-exposed RAW 264.7 cells.	[91]
Allicin	MTT assay Cell cycle by flow cytometry RT-PCR of *cyclin D1*, *MMP-9* and *RARβ*	CD44^+^ CD117^+^ cells	CD44+: 4–32 µg allicin/mL or 8–125 µg ATRA/mL or 5 µg/mL of allicin during 4 h followed by 8–125 µg ATRA/mL. Total time of exposure 48 h. CD117^+^: 0.5–24 µg allicin/mL or 4–64 µg ATRA/mL or 5 µg/mL of allicin during 4 h followed by 4–64 µg ATRA/mL. Total time of exposure 48 h. IC_50_ for 48 h.	IC_50_ CD44^+^: allicin/ATRA (17.53 µg/mL) ˂ allicin (29.19 µg/mL) ˂ ATRA (37.43 µg/mL) IC_50_ CD117^+^: ATRA (8.09 µg/mL) ˂ allicin (10.75 µg/mL) ˂ allicin/ATRA (13.65 µg/mL) ↑ of cells at the G2/M and G0/G1 phases in the CD44^+^ and CD117^+^ cells, respectively. The combination treatment caused the inhibition of CD44^+^ and CD117^+^ melanoma cells at the S phases compared to ATRA alone. ↑ cyclin D1 mRNA expression by all treatments and reduction of *MMP-9* mRNA expression by allicin treatment both CD44+ and CD117^+^ cells. ↑ mRNA level of *RARβ* expression by allicin/ATRA treatment in CD117^+^ cells. Increased *MMP-9* gene expression by allicin/ATRA and ATRA treatments in CD44^+^ cells. Allicin reinforces the ATRA-mediated inhibitory effects on CD44^+^ and CD117^+^ melanoma cells	[92]
DADS	Cell viability by trypan blue assay SiRNA Immunoblotting assay Apoptosis by flow cytometry DNA fragmentation assay Caspase-3/7 activity assay	HCT116 DLD-1 HT29 SW620 FHC	5–100 µM for 24 h. 0–25 µM 20 h + 50 ng/mL TRAIL for 4 h.	0–10 µM caused ˂20% CRC cell deaths. DADS + TRAIL produced concentration-dependent decreased of % survival in SW620 cells, but not in FHC cells. 0–10 µM did not alter the expression of pro-apoptotic proteins (Bax and Bid) or antiapoptotic proteins (XIAP and olorecta) and Bcl-2 were down-regulated in CRC cell lines.	[93]
Polymer films of *Allium cepa* L.	Cell viability by MTT assayAmes testMN assay	HepG2 GM-07492 *S. typhimurium* strains	Eluates from HTP-films and W-HTP films containing onion pulp were used at different concentrations	Cytotoxicity: HTP > W-HTP. No induction of MN was observed in both type of films, although the HTP films showed signs of mutagenicity in the Ames test.	[56]
Triploid onium *Allium cornutum* Clementi ex Visiani, 1842, and common onion *Allium cepa* L.	Proliferation assay by MTS DNA fragmentation assay PCR of p53, Bax, Caspase 3	Hela, HCT116, and U2OS human cancer cell lines	Serial dilutions of extracts from both *Allium* species (containing sulfides) were added to the 3 cell lines.	Antiproliferative effects of both species were reported in the three cell lines. They induced apoptosis in HeLa cells.	[94]
Allicin	Determination of LC50 DNA fragmentation assay	*Schistosoma mansoni*	Not revealed	LC_50_ = 315 µL/L No DNA fragmentation	[95]

### 3.4. Risk of Bias

Studies were considered to have a low, moderate or high risk of bias in terms of score out of 10. A moderate risk of bias was found in each of the 43 works chosen for the present systematic review. When reviewing the quality of selection, the studies show more limitations in “reproducibility” and “adequate statistical analysis” items. Full details are given in Table 3.

**Table 3:** Risk of bias for the methodological quality of studies reporting the toxicological evaluation in vitro of OSCs from *Allium* spp. 0: not reported; 1: not appropriately or clearly evaluated; 2: appropriately evaluated. M: medium (5–7); L: low (8–10); H: high (0–4).

Reference	Clear Objective	Well Characterized Product	Reproducibility of the Assay	Comparability	Adequate Statistical Analysis	Total	Risk of Bias	General Risk of Bias
[57]	2	2	1	2	0	7	3	M
[58]	2	2	1	2	0	7	3	M
[59]	1	2	1	1	2	7	3	M
[60]	2	2	2	1	1	8	2	L
[61]	2	1	1	2	2	8	2	L
[62]	2	2	1	1	2	8	2	L
[63]	2	0	2	2	2	8	2	L
[64]	2	2	2	2	2	10	0	L
[65]	2	1	2	2	2	9	1	L
[66]	2	2	2	1	0	7	3	M
[67]	2	2	1	1	2	8	2	L
[68]	2	2	2	2	2	10	0	L
[69]	1	1	2	2	0	6	4	M
[70]	2	2	1	2	0	5	5	M
[71]	2	0	2	2	2	8	2	L
[72]	2	2	1	2	2	9	1	L
[75]	1	2	1	2	2	8	2	L
[73]	2	2	2	2	2	10	0	L
[74]	2	2	1	2	2	9	1	L
[76]	2	1	1	2	0	6	4	M
[77]	2	0	2	2	2	8	2	L
[78]	2	2	2	2	1	9	1	L
[79]	2	2	0	2	2	8	2	L
[80]	2	2	1	2	2	9	1	L
[81]	2	2	2	2	0	8	2	L
[82]	2	0	1	2	2	7	3	M
[83]	2	2	1	1	2	8	2	L
[84]	1	2	1	1	2	7	3	L
[85]	2	2	2	1	2	9	1	L
[16]	2	2	2	2	2	10	0	L
[17]	2	2	2	2	2	10	0	L
[86]	2	2	1	1	2	8	2	L
[87]	2	2	1	2	2	9	1	L
[88]	2	2	2	2	2	10	0	L
[89]	2	2	2	2	0	8	2	L
[55]	2	2	2	2	2	10	0	L
[91]	2	2	2	1	2	9	1	L
[92]	2	1	1	1	2	8	2	L
[90]	2	2	2	2	2	10	0	L
[93]	2	1	1	2	0	6	4	M
[56]	2	0	2	1	2	7	3	M
[94]	2	2	2	2	2	10	0	L
[95]	2	2	0	0	0	4	6	H

### 3.5. Limitations

The present systematic review was restricted by the databases used, the search conditions, and the recognized inclusion/exclusion principles chosen. However, the exploration strategy was quite comprehensive, so it is expected that relatively, only a few important studies could not be identified and considered. Only works reported in English were included, and this point could indicate bias in the source searching and selection process [96]. Finally, the lack of sufficient statistical information made impossible to combine the results of different studies into a meta-analysis section that it had to be divided by subheadings. It should provide a concise and precise description of the experimental results, their interpretation, as well as the experimental conclusions that can be drawn.

### 3.6. In Vivo Studies Excluded

Several in vivo studies (*n* = 26, Figure 1), despite being excluded by the criteria of this systematic review, have been analyzed by the authors. As in in vitro studies, many of them deal with the anticancer properties of natural organosulfur compounds, mainly assayed in mice. In this sense, Sundaram et al. [97] studied the growth inhibitory properties of DADS against colon cancer, Chu et al. [98] studied the compound S-Allylcysteine against prostate cancer and Nishikawa et al. [99] studied the inhibitory properties of ajoene against skin cancer. Other in vivo studies focused on measuring biological markers, such as catalase and monooxygenase activity, or their protective properties against toxic substances in animals treated with alliaceous compounds. In this sense, Zhang et al. [100] studied the protective effect of allicin against acrylamide. Only a few in vivo studies have focused on evaluating the toxicity of alliaceous compounds. Thus, acute studies [20] or subchronic toxicity studies of isolated substances (such as PTSO) [101] or extracts from plants of the *Allium* genus [102] were found. Among the in vivo toxicological studies, the genotoxicity tests (MN and comet) in rats are highlighted [103,104]. In general, no significant signs of toxicity neither genotoxic effect were observed in the subchronically studies or genotoxicity endpoints.

## 4. Discussion

The beneficial effects of OSCs compounds have been reviewed by different authors. In addition, their phytochemical profile has been well described [5,12]. However, as far as we know, the safety evaluation and toxicity effects of these compounds have not been reported.

The number of scientific publications dealing with the in vitro toxicity of OSCs that meet the criteria established in this review amounts to 43. In vitro studies play an important role in the toxicity evaluation of compounds. They can give valuable hints about mechanisms of toxicity, providing rapid and cost-effective screening, and allow one to reduce the use of live animal models in research.

Among the OSCs investigated, most studies have focused on DADS, DATS and DAS, followed by DATS, DAS, Allicin and Ajoene, whereas for others, the existing reports were limited (i.e., DPS, DPDS, PTSO, PTS) (see Figure 2). Moreover, most of the toxicity studies of OSCs are reported from 1990s and early 2000s, and only 14 of them have been published after 2015, so there are few current toxicological studies focusing on the toxicological effects of these compounds.

The most frequently used assays included cell viability determination, mechanisms of cell death (apoptosis), cell cycle analysis, oxidative stress biomarkers, mitochondria membrane potential (MMP), gene expression by PCR and protein expression by Western blotting. Cytotoxicity has been tested mainly by the MTT test [56,57]. Apoptosis has been investigated mainly by flow cytometry [65,93], but also by microscopic evaluation [63,66,70,80,84,86,93]. Moreover, expression of related genes and proteins (such as Bcl-2, Bax, p53, etc.) has also been explored [63,68,94] as well as caspases activity [68,74,80,87,93], but to a lesser extent. 

Most of these studies aimed to evaluate the cytotoxicity of OSCs on different cellular models and tried to elucidate the mechanisms involved. Indeed, the vast majority of them explored the antiproliferative effects of OSCs to justify their potential as chemoprotectants against carcinogenesis (see Table 2). Several studies that reported the anti-cancer effect of black garlic on the cancer cell line showed inhibition of tumor activity by regulating metabolism [12]. Furthermore, DADS has been proposed as a therapeutic strategy for oxidative stress-injury in neurodegenerative diseases [65] and DAS has proved to be effective in reducing ethanol induced injury of cells (Kema et al., 2018). Apart from therapeutic aspects, there are a limited number of papers dealing with genotoxicity [16,17,55,56,60,62,95]. Finally, only 4 out of 43 studies have been performed specifically to explore the safety of OSCs for further agrifood applications [16,17,55,56]

Regarding results obtained for specific OSCs, DADS in relation to genotoxicity aspects has been reported to induce chromosome aberrations and sister chromatid exchanges in a Chinese hamster’s ovary cell line (CHO) [60]. Additionally, it increased the mutagenicity of 2-amino-1-methyl-6-phenylimidazol [4,5-b] pyridine when the Ames test was performed with S9 fraction from rats exposed to DADS. Filomeni et al., [64] and Kim et al. [65] explored the effects of DADS on two different cellular models of the nervous system and obtained different results. Whereas the first one suggested a pivotal role for oxidative stress in DADS-induced apoptosis on SH-SY5Y cells and pointed out a potential use as antiproliferative agent in cancer therapy, the second one observed opposite results on N18D3 cells, depending on the concentration used, with a protective effect at low concentrations. Recently, important toxic effects have been reported of this compound associated to high doses [105]. For these reasons, more DADS toxicity studies are necessary to guarantee its safe use as an anticancer agent.

Two other studies [70,75] investigated its effects on a breast cancer cell line (MCF-7) and obtained similar results, inhibition of cell proliferation and apoptosis induction, with Lei et al. [70] providing mechanistic clues (inhibition of ERK and activation of SAPK/JNK and p38 pathways). Apoptosis was also observed in other different cell types where the antiproliferative effects of DADS were investigated such as p53-wild type H460 and p53-null type H1299 non-small-cell lung cancer cells [63], in human glioblastoma cells [68], human leukemia cells [72], human esophageal carcinoma cells [84,85] or primary effusion lymphoma cells [87]. All these reports support the potential use of DADS as chemotherapeutic agent.

Results reported for DATS are similar to those discussed for DADS, as DATS have also shown to induce cytotoxicity, ROS production or apoptosis (also evidenced by changes in the expression of related genes and proteins) in different cell types such as NIH-3T3, MCF-7, PC-3, AGS, U87M6, PNT-1A, MDA-MB468, MDA-MD231, A172, U343 and T98G. Only Das et al. [68] and Shigemi et al. [87] evaluated both DADS and DATS, and compared the results obtained. Both of them observed similar results: DATS was more potent than DADS and DAS for induction of cell death with involvement of mitochondria and ROS production.

With respect to DAS, it induced genotoxic effects similarly to DADS [60,62], but it was less cytotoxic to CHO cells and it increased the activation of a higher number of mutagens. Again, several studies in a variety of cell lines showed its antiproliferative effect [59,63,68,71,77,87]). It has been reported that garlic compounds (DAS, DADS, DATS) do not require a p53-dependent pathway for mediation of apoptosis [68]. Moreover, its potential to reduce the tissue injury caused by ethanol was also demonstrated [92]. In order to compare the effects produced by each of these compounds, differences have been found, mainly due to the diverse experimental models and conditions used (concentration, time of exposure, biomarkers, etc.).

Studies evaluating allicin mainly reported cytotoxicity [57], and apoptosis [66,83,86,90] as adverse outcomes, mediated by different key events such as altered genes and protein expression or cell cycle changes. Scharfenberg et al. [57] were the only ones that studied not only allicin but also its decomposition product ajoene and observed that allicin was less toxic than ajoene in three different cell lines. This compound, ajoene, was also investigated by different authors [58,67,73] that observed cytotoxicity (with different cell lines showing different sensitivity) and apoptosis. 

Regarding cytotoxicity assays, there are only a few cell lines listed in these studies that are included in the guidelines by OECD guidance for toxicological evaluation of chemicals. Most of them are cancer cell lines, and the effects reported in these findings correspond to a therapeutic anticancer effect and not to a cytotoxic evaluation. In this sense, more studies focused on the toxic effect of OSCs isolated are necessary.

Moreover, those OSCs with the fewer number of studies available in the scientific literature were also those mainly focused on safety issues in relation to their use in the agri-food sector. Thus, neither mutagenicity by the Ames test nor cytotoxicity in the human intestinal carcinoma Caco-2 cell line was observed for DPS and DPDS [16]. Additionally, a complete battery of genotoxicity tests were performed for PTSO [17] and PTS [55].

Genotoxicity assessment plays a key role in the safety evaluation required by EFSA guidelines for the submission of dossiers of different substances, such as food and feed additives, etc. [48,50], with the basic battery performed with in vitro tests. However, results of this review showed that only few assays have been carried out and some studies do not include the basic battery of tests required by the EFSA. In this regard and taking into account that specific OSCs that have been mainly investigated for their chemotherapeutic potential such as DADS, DATS, DAS, etc., show also interesting activities for their use in the food industry (antimicrobial, antioxidant or antifungal activities, among others, see Table 1), the thorough study of their genotoxicity would be worthy of research. Moreover, advanced in vitro models (i.e., 3D) could provide new data to support in vitro-in vivo data extrapolation for OSCs in general, and the testing of relevant concentrations used in the agri-food sector would allow to consider both efficacy and safety aspects. Thus, in vitro assays on their own can still provide valuable information to contribute to the commercial use of OSCs.

## 5. Conclusions

In general, there are very few in vitro studies focused on investigating the potential toxicity of OSCs. Most research studies aimed at evaluating only the cytotoxicity of OSCs on different cellular models to elucidate antiproliferative effects of these compounds and justify their potential as chemoprotective agent against carcinogenesis. This makes it difficult to assess the safety of the use of these compounds for a correct risk assessment. In addition, it limits the preliminary information needed to proceed with an in vivo toxicity assessment. Therefore, other cellular models such non-cancer cell lines should be included to ensure a correct in vitro toxicity evaluation of these compounds. Specifically, considering that genotoxicity assessment plays a key role in the safety evaluation required by EFSA; more genotoxicity studies of OSCs are necessary to guarantee consumer safety before their use as a potential natural additive in the food industry.

## Figures and Tables

**Table 1:** Organosulfur compounds present in *Allium* species, chemical structure and main properties.

Name	Chemical Structure	Mode of Action	Reference
E-Ajoene Z-Ajoene	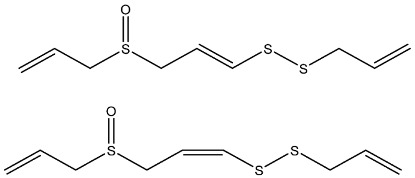	Antibacterial: in vitro activity against Gram +: MIC = 5–250 µg/mL and Gram –: MIC = 100–500 µg/mL	[21]
		Antifungal: MIC = 15–50 µg/mL	
		Antioxidant enzyme induction: NAD(P)H: quinone oxidoreductase-1 (NQO1)	[22]
Alliin	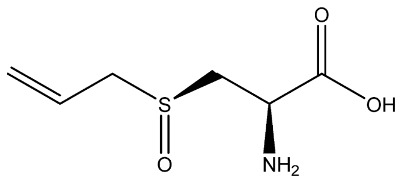	Antioxidant: superoxide scavenging activity	[23]
Allicin	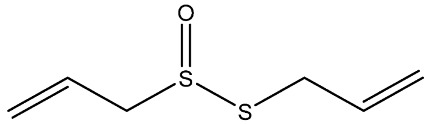	Antibacterial: in vitro activity against *Staphylococcus* strains MIC = 600 µg/mL and Gram-bacteria: MIC = 4–300 µg/mL	[24,25,26]
		Antifungal: MIC = 1.52–6.25 µg/mL	[27]
		Antioxidant activity in vivo: SOD and GSH-Px activities increased. Radical scavenging ability of hydroxyl radical increase with Allicin	[24,28,29]
Di-allyl-disulphide (DADS)	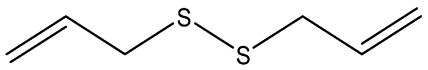	Antibacterial activity against *S. aureus*: MIC = 4 µg/mL and *Helicobacter pylori* MIC = 200 µg/mL	[25,30]
		Antifungal activity against *Aspergillus* spp: MIC = 8–12 µg/mL and *Candida* spp: MIC = 4–12 µg/mL	[30]
		Antioxidant in vivo activity	[31,32]
Di-allyl-sulphide (DAS)	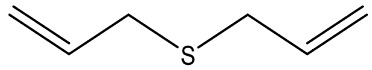	Antibacterial activity against *S. aureus*: MIC = 20 µg/mL and *H. pylory* MIC = 4 µg/mL	[25,30]
		Antifungal activity against *Aspergillus* spp: MIC= 40–64 µg/mL and *Candida* spp: MIC= 32–72 µg/mL	[30]
		Antioxidant in vivo activity	[33]
Di-allyl-trisulphide (DATS)	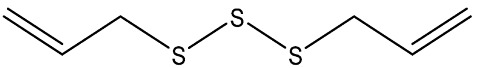	Antibacterial activity against *S. aureus*: MIC = 2 µg/mL and *H. pylory* MIC = 25 µg/mL	[25,30]
		Antifungal activity against *Aspergillus* spp: MIC = 2–8 µg/mL and *Candida* spp: MIC = 1–8 µg/mL	[30]
		Antioxidant in vitro activity	[34]
Dipropyl disulphide (DPDS)	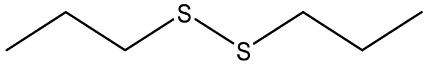	Antioxidant in vitro activity	[16]
Dipropyl sulphide (DPS)	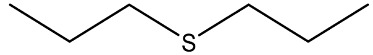	Antioxidant in vitro activity	[16]
Propyl-propane-tiosulphonate (PTSO)	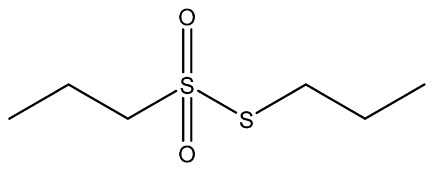	Antibacterial activity against Gram +: MCB = 0.5–10 µg/mL and Gram-: MCB = 1.25–10 µg/mL	[20,35,36]
		Antifungal: MFC against *Verticillium dahliae* = 19.53–39.06 µg/mL MFC_90_ against *Candida* spp. = 64–128 µg/mL	[11,37]
		Antiprotozoal and Antiparasitic: reduce the number of apicomplexa in monogastric animals. Reducing a plurality of aquatic parasites in aquatic animals.	[38,39]
		Antioxidant	[20]
Propyl-propane-tiosulphinate (PTS)	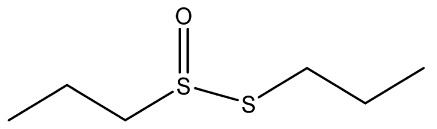	Antibacterial activity against Gram -: MCB = 128–1024 µg/mL and Gram +: MCB = 128 µg/mL	[11]
		Antifungal activity MFC against *Verticillium dahliae* = 78.13 µg/mL MFC_90_ against *Candida* spp = 128 µg/mL	[34,40]
		Antiprotozoal and antiparasitic: reduce the number of Apicomplexa in monogastric animals. Reducing a plurality of aquatic parasites in aquatic animals.	[38,39]
S-allylcysteine (SAC)	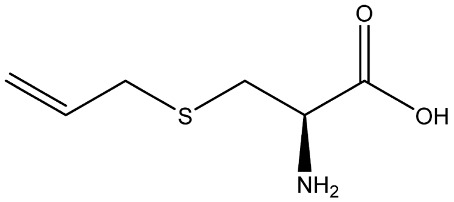	Antioxidant activity by scavenging ROS	[41]
Vinyldithiin	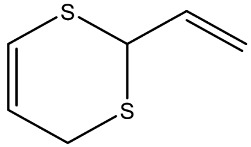	Antioxidant activity	[42]

## Data Availability

The data presented in this study is contained within the article.

## Abbreviations

3T3-HER2: Mice fibroblast cell line; 3T3-L1: Mice fibroblast cell line; 5-Fu: 5-fluorouracil; A172: Human brain cell line; A375: Human melanoma cells; A549: Adenocarcinomic human alveolar basal epithelial cells; AGS: Human gastric carcinoma cell line; AIF: Apoptosis inducing factor; AMPK: AMP-activated protein kinase; ALP: Alkaline phosphatase activity; Asp: Aspartate; ATRA: All-trans retinoic acid; B16/BL6: Melanoma mouse cell line; B16F10: Murine melanoma cell; BC2: Human hepatoma cell line; BC3: Primary effusion lymphoma cell line; BCBL1: Human lymphoma cell line; BGC-823: Human gastric cancer cell line; BHK21: Baby hamster kidney-derived cell line; BJA-B: Burkitt lymphoma-derived cell line; Caco-2: Human colorectal adenocarcinoma cell line; CHO: Chinese hamster ovary cells; CRC: Human colon cancer cell line; CTX: Cyclophosphamide; Cyt c: Cytochrome c; DADS: Diallyl disulfide; DAS: Diallyl sulfide; DATS: Diallyl trisulfide; DLD-1: Colorectal adenocarcinoma cell line; DG75: Human lymphoma cell line; DPS: Dipropyl sulphide; DPDS: Dipropyl disulfide; ECA109: Human esophageal carcinoma cell line; ED50: amount of test compound required to reduce cell viability to 50%; ERK: Stress-activated protein kinase extracellular signal-regulated kinase; ERK-2: Extracellular regulatory kinase-2; EROD: Ethoxyresorufin O-deethylase; FHC: Normal human colon cell line; FS4: Human foreskin-derived cell line; GGPP: Geranylgeranylpyrophosphate; Glu: L-glutamate; GM-07492: Primary human skin fibroblasts; GSH: Reduced Glutathione; GSH-Px: Glutathione peroxidase; GSK-3: Glycogen syntethase kinase-3; GSSG: Oxidized glutathione; HBL6: Human lymphoma cell line; HEL: Human erythroleukemia cell line; HeLa: Cervical cancer cell line; HepG2: Human liver cancer cell line; HL-60: Human leukemia cell line; HT29: Human colorectal cell line; HTC116: Human colon cancer cell line; Http: Unwashed hydrothermally treated pulp; JNK/c-Jun: c-jun terminal kinase; M14: Human melanoma cell line; MAPKs; Mitogen-activated protein kinases; MCB: Minimal Bactericidal Concentration; MCF-7: Human breast cancer cell line; MDA: Malondialdehyde; MDA-MB-231: Human breast cell line; MDA-MB-468: Human breast cell line; MFC: Minimum fungicidal concentration; MIC: Minimal Inhibitory Concentration; MGC-803: Human gastric cancer cell line; MLA: Mouse lymphoma thymidine-kinase assay; MMP: Mitocondrial membrane potential; MN: Micronucleus; MROD: Methoxyresorufin O-demethylase; MTS: 3-(4,5-dimethylthiazol-2-yl)-5-(3-carboxymethoxyphenyl)-2-(4-sulfophenyl)-2H-tetrazolium, inner salt; NF-κB: Nuclear factor enhancing the kappa light chains of activated B cells; NIH-3T3: Mice fibroblasts cell line; NR: neutral red; L02: Human normal liver cell line; LDH: Lactate dehydrogenase activity; LEC1: Leuco-phytohemagglutinin resistant cell line; OCIM-1: Human leukemia cell line; p38: Mitogen-activated protein kinase; PAGE: Protein extracts and polyacrylamide gel electrophoresis; PANC-1: Human pancreatic carcinoma; PC: Protein content; PC-3: Human prostate adenocarcinoma cell line; PI3K/Akt: Phosphatidylinositol 3-kinase; PNPH: p-Nitrophenol hydroxylase; PROD: pentoxyresorufin O-dealkylase; PTS: Propyl-propane thiosulphinate; PTSO: Propyl-propane thiosulphonate; ROS: Reactive oxygen species; Ramos: Epstein-Barr virus-negative Burkitt lymphoma cell line; RAW 264.7: Mouse macrophage cell line; ROS: reactive oxygen species; RT-PCR: Quantitative realtime polymerase chain reaction; SAPK: stress-activated protein kinase; SCEs: sister chromatid exchanges; SGG-7901: Human gastric cancer cell line; SiRNA: small interfering RNA; SKBR-3: Human breast cancer cell line; SOD: Superoxide dismutase; SW620: Human colorectal cancer cell line; T98G: Glioblastoma cell line; TRAIL: Tumor necrosis factor-related apoptosis-induced ligand; U251: Human glioma cell line; U2OS: Human osteosarcoma cell line; U343: Human glioblastoma cell line; U87MG: Human glioblastoma cell line http:HTP: Washed hydrothermally treated pulp.
